# Mechanically stable X_2_InAsF_6_ (X = K, Rb, Cs) for optoelectronic and wasted heat recovery applications

**DOI:** 10.1039/d6ra07016g

**Published:** 2026-10-12

**Authors:** Nimra Asif, Muhammad Yaseen, Nada Alfryyan, Sidra Sarfaraz, Muhammad Adnan, Imed Boukhris

**Affiliations:** a Department of Physics, University of Agriculture Faisalabad Faisalabad 38040 Pakistan myaseen_taha@yahoo.com; b Department of Physics, College of Sciences, Princess Nourah bint Abdulrahman University P.O. Box 84428 Riyadh 11671 Saudi Arabia; c Central Labs, King Khalid University AlQura'a, P.O. Box 960 Abha Saudi Arabia; d Department of Physics, Faculty of Science, King Khalid University P.O. Box 960 Abha Saudi Arabia

## Abstract

Herein, the structural, elastic, optoelectronic and thermoelectric properties of double halide X_2_InAsF_6_ (X = K, Rb, Cs) perovskites are investigated using density functional theory (DFT). The analysis of structural properties indicates that the studied compounds adopt a stable non-magnetic ground state with *Fm*3̄*m* symmetry. The calculated octahedral factor (Δ*µ*), Goldschmidt tolerance factor (*τ*_G_), modified tolerance factor (*τ*), and formation enthalpy (Δ*H*_f_) confirm their structural and thermodynamic stability. Mechanical parameters, including Poisson's ratio (*ν* > 0.26), Pugh's ratio (*B*/*G* > 1.75), and the anisotropy factor (*A* ≠ 1), confirm that all compounds are ductile and elastically anisotropic. The modified Becke Johnson potential (mBJ) calculations reveal direct band gaps of 2.61 eV (K_2_InAsF_6_), 2.70 eV (Rb_2_InAsF_6_), and 2.74 eV (Cs_2_InAsF_6_). Optical analysis reveals strong absorption extending from the visible to ultraviolet regions with high absorption coefficients (*α* ≈ 10^4^ cm^−1^) in the 5.9–6.4 eV energy range. Furthermore, the investigated compounds exhibit high Seebeck coefficients (>200 µV K^−1^) and figures of merit (>0.7) throughout the 300–800 K temperature range. Overall, these findings demonstrate the significant potential of X_2_InAsF_6_ compounds for optoelectronic and energy conversion technologies.

## Abstract 2

The mBJ potential calculations reveal direct *E*_g_ of 2.61/2.70/2.74 for K_2_InAsF_6_/Rb_2_InAsF_6_/Cs_2_InAsF_6_, respectively. Furthermore, investigated compounds exhibit high figures of merit (>0.7) within 300–800 K.

## Graphical abstract

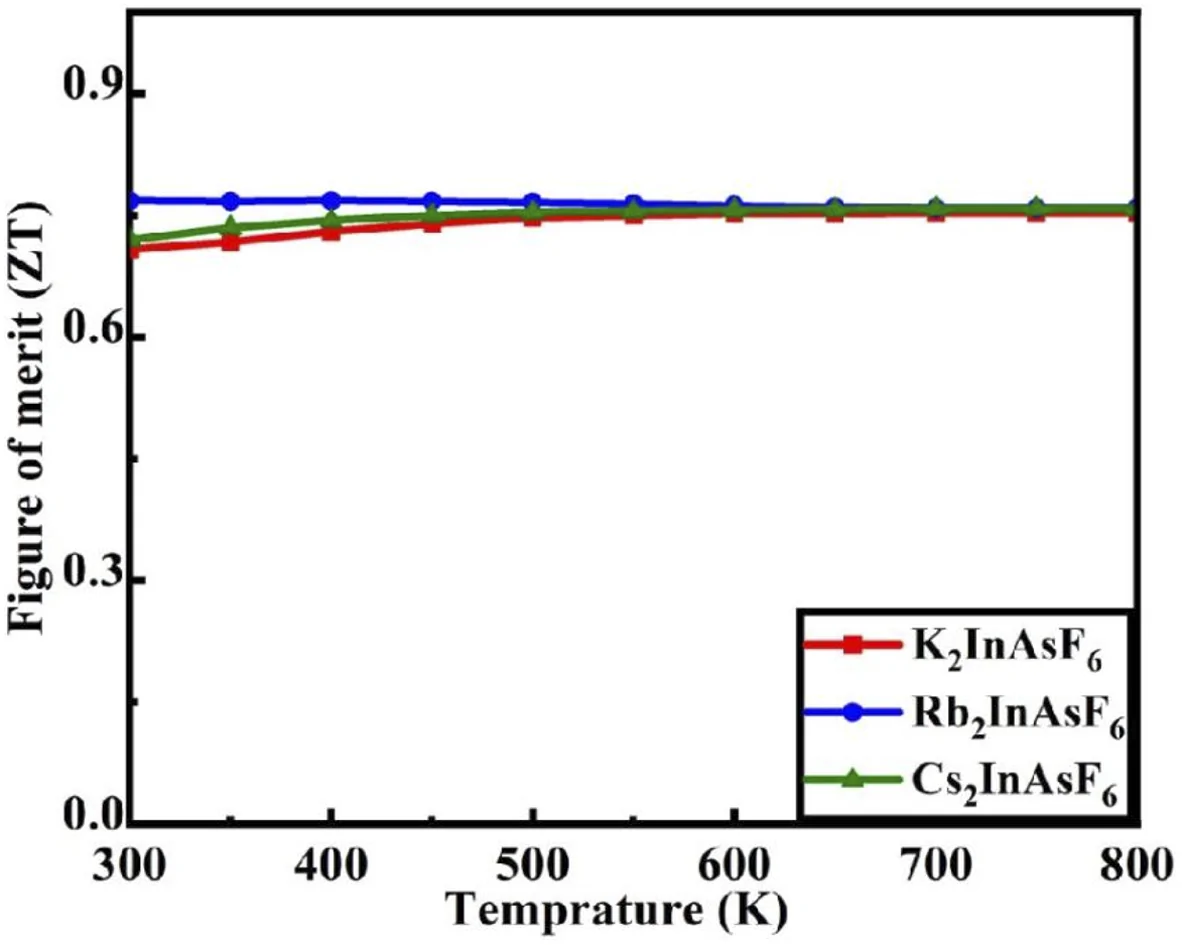

## 1. Introduction

Over the past few decades, increasing energy demand, environmental concerns, and continuous depletion of traditional fossil fuels have boosted the search for green energy resources. Traditional energy resources, including coal and petroleum, are not only major contributors to greenhouse gas emissions but are also being exhausted at an alarming rate, which makes the development of renewable energy alternatives a global priority. In this regard, significant research attention has shifted toward renewable energy systems, particularly thermoelectric (TE) systems that directly transform heat into electrical energy and photovoltaic (PV) systems that efficiently convert solar radiation into electricity.^1,2^ Among various energy conversion approaches, the TE technology provides an eco-friendly pathway for conversion because the systems operate without the need for moving mechanical parts. However, despite significant research efforts, achieving high TE conversion efficiency remains the principal challenge due to the strong interdependence of thermal and electrical transport features.^3^

Traditional and advanced TE materials including Bi_2_Te_3_,^4^ AgSbTe_2_,^5^ GeTe,^6^ Pb_2_FeSbO_6_,^7^ Cs_2_AgBiBr_6_,^8^ and PbTe^9^ demonstrate excellent thermoelectric performance. However, their large-scale application is hindered by poor thermal stability, toxicity, scarcity, and high manufacturing costs. Consequently, increasing interest has been directed toward the materials that can be synthesized through simple, cost-effective, and environmentally friendly methods.

In the field of renewable energy, perovskite materials are one of the promising candidates owing to their versatile nature and tuneable physical properties.^10^ In particular, double perovskites have gained considerable research focus since their opto-electronic properties and thermal transport properties can easily be modified *via* chemical substitutions. Due to this compositional flexibility, the limitations associated with conventional TE and optoelectronic materials can be mitigated. The cation substitution provides an effective strategy for optimization of key parameters including carrier transport, lattice dynamics, optical responses and electronic band structures. In addition, the inherent structural complexity of DHPs can promote phonon scattering, which may contribute to reducing lattice thermal conductivity, an essential requirement for efficient TE energy conversion. These properties, coupled with the growing availability of lead-free compositions, provide further opportunities for developing environmentally benign and readily tunable functional materials.^11^

Double perovskites generally adopt the formula A_2_BB′X_6_, in which A denotes alkali/rare earth elements, B/B′ are different metal ions, and X could be oxygen, halides, or hydrides.^12–14^ The DHPs have emerged as possible candidates for optoelectronic devices including photovoltaics, radiation sensors, photodetectors, and lasers. The increasing interest in DHPs has led to extensive experimental and theoretical studies aiming at evaluating their potential for large-scale applications. Experimentally, the Na_2_AgBiBr_6_ compound has been successfully synthesized and reported to exhibit suitable UV-VIS absorption spectra, enabling them for TE and optoelectronic systems.^15^ In parallel, DFT calculations have been generally used to probe magnetic, optoelectronic, and transport features of cubic DHPs.^16–19^ A variety of lead-free DHPs has been thoroughly examined with respect to photovoltaic applications, which include Ba_2_AgBiM_6_ (M = I, Br, and Cl),^20^ Cs_2_NaInM_6_ (M = Br, Cl, and I)^21^ and Na_2_ReAlO_6_.^22^ Besides application in photovoltaic devices, DHPs have recently drawn considerable interest in the field of TE materials owing to their low lattice thermal conductivity. For instance, K_2_XSbCl_6_ (X = In, Au) has been reported to exhibit good TE performance with a value of *ZT* > 0.7.^23^ Similarly, Khan *et al.* have studied mechanical, optical and TE features of Rb_2_YCuCl_6_ and Cs_2_YCuCl_6_ materials. Band gap energies of these materials have been found to be 2.57 eV and 2.39 eV for Rb_2_YCuCl_6_ and Cs_2_YCuCl_6_, correspondingly.^24^ Despite the growing demand of research on DHPs, most studies have predominantly focused on chloride- and bromide-based compounds, while fluoride-based DHPs, such as X_2_InAsF_6_ (X = K, Cs, Rb), have not received much attention. To date, a systematic investigation of their physical properties has not yet been reported. To address the above mentioned gap, the present work employs DFT-based calculations within the Wien2k code to comprehensively examine the X_2_InAsF_6_ DHPs family. This research elucidates the impacts of A-site replacement on elastic robustness, structural firmness, optical response, electronic properties and TE performance of fluoride-based double perovskites. The obtained results establish the suitability of these lead-free DHPs for next-generation high-performance optoelectronic and energy-harvesting usages.

## 2. Computational details

The various physical properties of X_2_InAsF_6_ DHPs were determined using the DFT-based full potential linearized augmented plane wave method (FP-LAPW) within the Wien2k code.^25,26^ This quantum-mechanical has emerged as one of the most consistent tools for successfully computing the optoelectronic features of various crystalline solids.^27–29^ The exchange correlation effects were treated through the generalized gradient approximation (GGA) combined with mBJ to achieve accurate description of electronic properties.^30,31^ In FP-LAPW scheme, the crystal splits into an interstitial region (IR) and a non-overlapping muffin-tin (MT) surrounding the atomic sites. The plane waves are employed in the IR, whereas the electronic wave functions inside the MT spheres are expanded in spherical harmonics up to a maximum angular momentum of *l*_max_ = 10.0. The plane-wave basis set is defined by a cutoff parameter of *R*_MT_ × *K*_max_ = 8.0, while Fourier charge density was calculated using *G*_max_ = 14.0 (a.u.)^−1^.^32^ The adopted *R*_MT_ values were 2.50, 2.50, 2.50, 2.4, 1.95, and 1.95 Bohr, respectively, for the K, Rb, Cs, In, As and F atoms. An energy convergence criteria of 10^−5^ Ry was used to achieve self-consistency. A dense k-mesh comprising of 1000 points was employed to sample the first Brillouin zone (BZ) to accurately determine the periodic nature of crystal structure. The TE properties were evaluated using the BoltzTrap algorithm, while the elastic properties were subsequently determined through IRelast package.^33,34^

## 3. Results and discussions

### 3.1 Structural properties

A detailed evaluation of structural features is vital for comprehension the atomic configuration and stability of crystalline solids. The X_2_InAsF_6_ (X = K, Rb, Cs) DHPs adopt cubic *Fm*3̄*m* symmetry, in which X site cations (X = K, Rb, Cs) occupy the 8c (0.25,0.25,0.75) Wyckoff positions, while In and As atoms are located at 4b (0.5,0,0) and 4a (0,0,0) sites, respectively. The halide ion F resides at the 24e (0.223,0,0) Wyckoff positions, forming the characteristic frame work of DHPs. The optimized unit cell lattices and corresponding energy-volume optimization graphs are presented in Fig. 1(a–i). The ground-state properties including lattice constants are compiled in Table 1 and compared with published work data for structurally analogous compounds. The calculated results are comparable with experimental and computational findings, indicating the consistency of present structural computations. The structural optimization was carried by varying unit-cell volume for both non-magnetic (NM) as well as ferromagnetic phases (FM) and fitting the resulting *E*–*V* data to Birch–Murnaghan equation of states which can be expressed as:^41^1
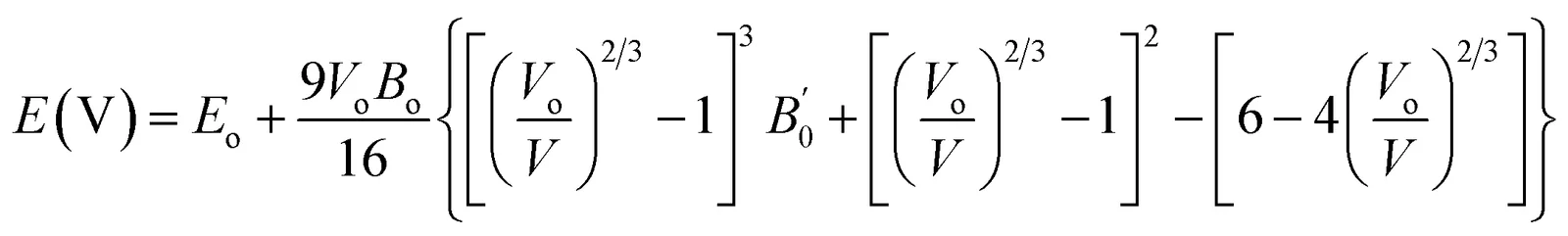


**Fig. 1:**
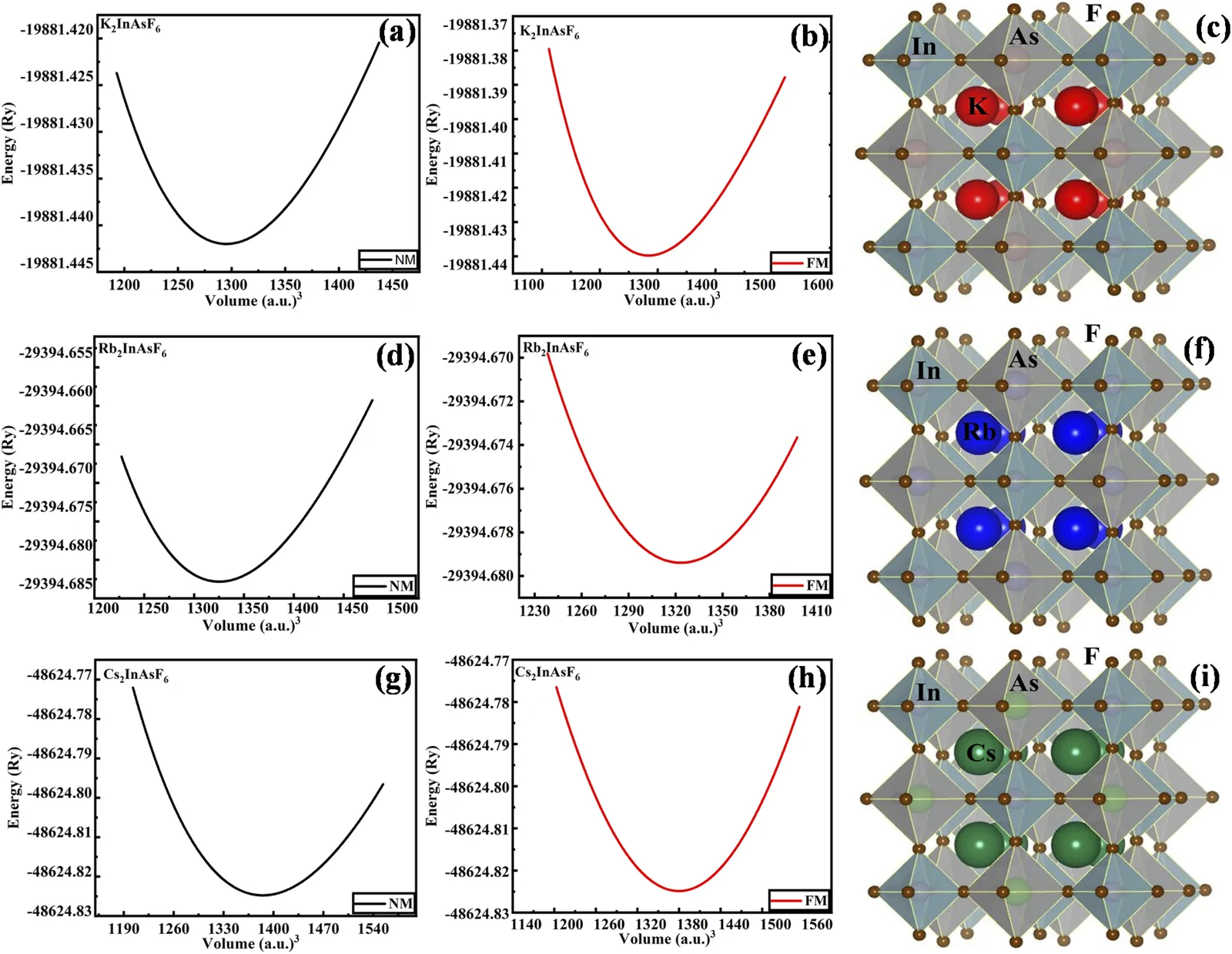
*E*–*V* curves of NM, FM and octahedral structure (a–c) for K_2_InAsF_6_ (d–f) for Rb_2_InAsF_6_ (g–i) for Cs_2_InAsF_6_.

**Table 1:** Structural parameters of K_2_InAsF_6_, Rb_2_InAsF_6_ and Cs_2_InAsF_6_

Structural parameters	K_2_InAsF_6_ (*Fm*3̄*m*)	Rb_2_InAsF_6_ (*Fm*3̄*m*)	Cs_2_InAsF_6_ (*Fm*3̄*m*)	K_2_InAgF_6_/Rb_2_InAgF_6_/Cs_2_InAgF_6_	K_2_NaInF_6_/Rb_2_KInF_6_/Cs_2_NaInF_6_
	This study			Previous study	
				Theoretical	Experimental
*a*_0_ (Å)	9.1561	9.2267	9.3634	9.05/9.12/9.12 (ref. 35)	8.56/9.09/8.90 (ref. 38–40)
*V*_0_ (a.u.)^3^	1295.0039	1325.1661	1384.9557	—	—
*B*_0_ (GPa)	53.6159	53.1383	49.8961	—	—
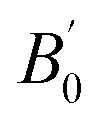	7.0501	7.4032	4.5438	—	—
*E*_0_ (Ryd.)	−19881.44	−29394.68	−48624.83	—	—
*τ*_G_	1	0.99	0.99	—	—
*µ̄*	0.517	0.516	0.518	—	—
Δ*µ*	0.08	0.08	0.08	—	—
*τ*	3.67	3.65	3.64	—	—
Δ*H*_f_ (eV)	−3.073	−3.108	−3.124	−2.29/−1.84/−1.94 (ref. 35–37)	—

The calculated curves reveal that all three compounds are energetically favourable in the NM phase as the minimum total energy is attained in NM configuration.

The structural firmness of studied DHPs was initially examined using different established stability indicators. The DHPs with the general composition A_2_BB′X_6_, preferentially adopt a cubic structure. However, distortion may arise due to certain factors including ionic size mismatch, Jahn–Teller effects, and octahedral tilting. Consequently, Goldschmidt tolerance factor (*τ*_G_),^42^ octahedral factor (*µ̄*),^43^ and octahedral mismatch (Δ*µ*), was calculated using the following mathematical relations:2
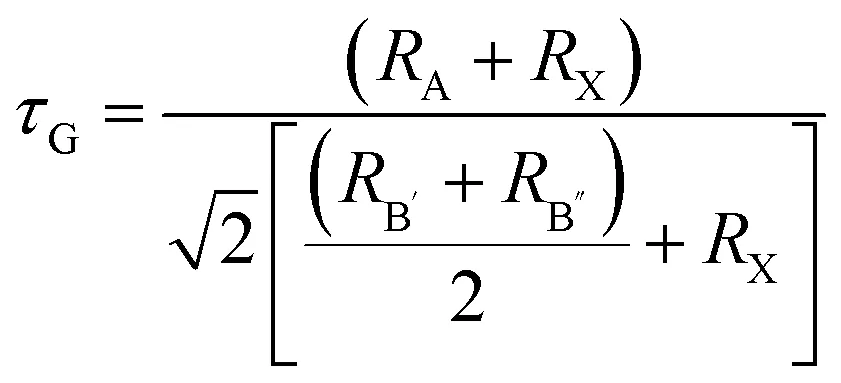
3
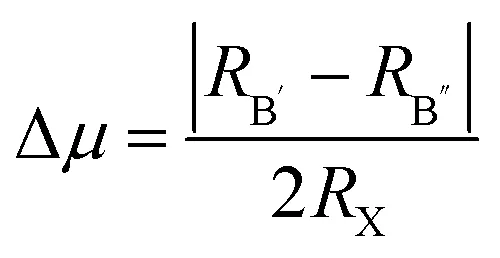
And4
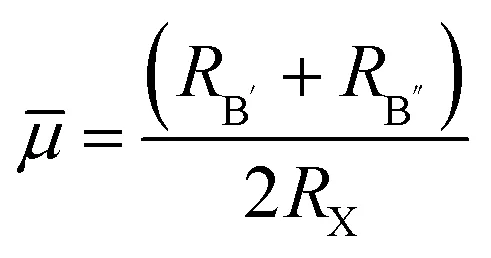


The tolerance factor assesses the geometric compatibility of the ions occupying the lattice sites, whereas, octahedral factor evaluates the firmness of BX_6_ octahedra. For a DHP material to be theoretically formable and structurally stable, the *τ*_G_ and *µ̄* must satisfy the conditions 0.81 ≤ *τ*_G_ ≤ 1.0 and 0.41 ≤ *µ* ≤ 0.90. Octahedral mismatch (Δ*µ*) describes the octahedral distortion arising from ionic size mismatch; a lower Δ*µ* value indicates that perovskite structure will be more stable. Furthermore, Bartel *et al.* recently proposed a modified tolerance factor (*τ*), that has been reported as more reliable predictor of structural stability, achieving an accuracy of 92%.^44^ The following mathematical expression is used to compute *τ*:5
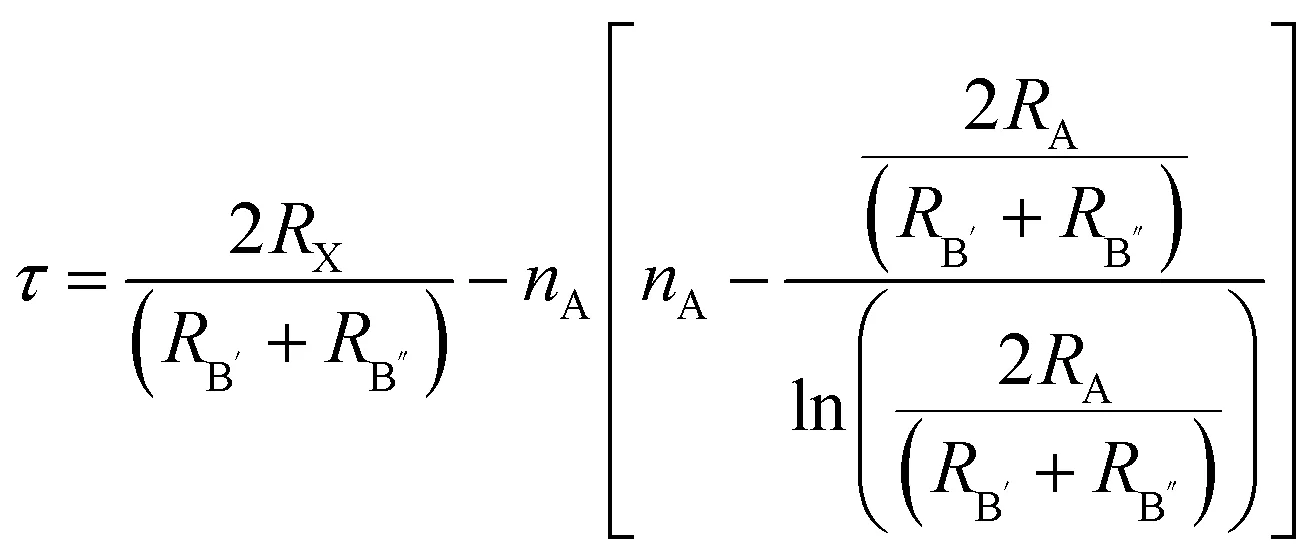


Generally perovskite compound are considered stable when *τ* < 4.18. The computed values of *τ*, *µ*, Δ*µ*, and *τ*_G_ are shown in Table 1. Observation of the results listed in table suggests that the values of all above parameters for X_2_InAsF_6_ lie within the limit necessary for a stable cubic structure.

The thermodynamic stability of studied DHPs, the formation enthalpy (*H*_f_) was evaluated through the following relation:^45^6
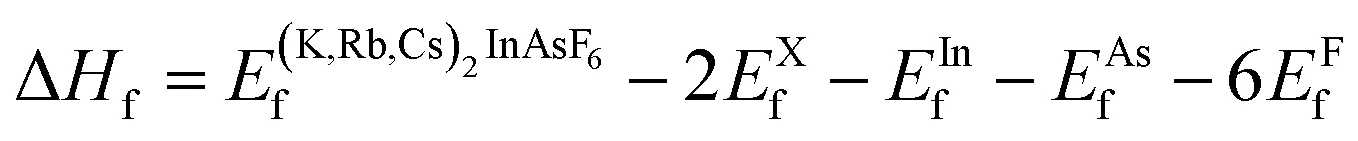


The computed values of Δ*H*_f_ are −3.073, −3.108 and −3.124 eV for K_2_InAsF_6_, Rb_2_InAsF_6_ and Cs_2_InAsF_6_, respectively. The negative value of Δ*H*_f_ suggests that the studied DHPs are experimentally synthesizable.

### 3.2 Elastic properties

Elastic properties are vital in evaluating their suitability for electronic and energy conversion devices, where materials with superior elastic performance are required. The mechanical behavior of a system is strongly governed by its elasticity, which is quantified through elastic constants. For cubic systems, three elastic constants namely, *C*_11_, *C*_12_, *C*_44_ are adequate to completely describe their mechanical behavior.^46^ The calculated results of *C*_11_/*C*_12_/*C*_44_ are 77.95, 24.11, and 12.64 GPa for K_2_InAsF_6_; 79.94, 20.17, and 15.07 GPa for Rb_2_InAsF_6_; 69.55, 22.76, and 11.03 GPa for Cs_2_InAsF_6_, respectively. The *C*_11_ and *C*_12_ describe the directional variations in elastic parameters associated to axis, while *C*_44_ measures the opposition of a substances to shape distortion under exterior applied pressure.^47^ Among all these studied compounds, Rb_2_InAsF_6_ exhibits the highest *C*_11_ and *C*_44_, representing greater resistance to longitudinal and shape deformation. The mechanical firmness of studied materials is ensured through the Born–Huang stability conditions based on computed elastic constants, as summarized in Table 2. For cubic lattices, the conditions *C*_11_ − *C*_12_ > 0, *C*_11_ + 2*C*_12_ > 0 and *C*_11_, *C*_44_ > 0 must be fulfilled.^48^ The X_2_AsInF_6_ compounds fulfill these conditions, confirming their stability.

**Table 2:** Elastic parameters of K_2_InAsF_6_, Rb_2_InAsF_6_ and Cs_2_InAsF_6_

Parameters	K_2_InAsF_6_	Rb_2_InAsF_6_	Cs_2_InAsF_6_
*C*_11_	77.950613	79.949784	69.551759
*C*_12_	24.116405	20.176535	22.767543
*C*_44_	12.647265	15.077432	11.035817
*G*_V_ (GPa)	18.35	20.00	15.9783
*G*_R_ (GPa)	16.05	18.17	13.9922
*G* (GPa)	17.20	19.09	14.99
*B* (GPa)	42.06	40.10	38.36
*E* (GPa)	45.41	50.18	39.78
*B*/*G*	2.44	2.10	2.56
*ν*	0.32	0.314	0.327
*A*_Z_	0.47	0.504	0.472
*C*_P_	11.47	5.10	11.73
*T*_m_ (K)	1014	964	1026

Different elastic terms such as bulk modulus (*B*), Zener anisotropy factor (*A*_Z_), and Young's modulus (*E*) are determined by using the following relations:^49^7
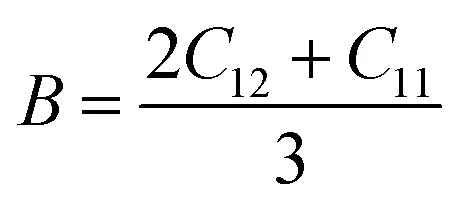
8
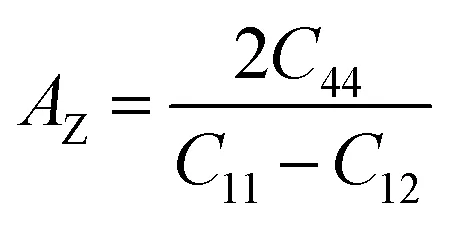
9
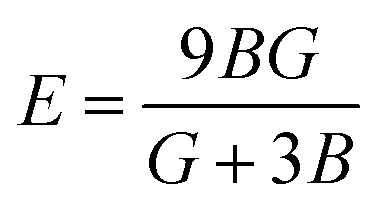


To further evaluate their mechanical stability some additional parameters including Cauchy pressure (*C*_P_), Poisson's ratio (*ν*) and shear modulus (*G*) are also calculated using Voigt–Reuss–Hill scheme:^50–52^

10 *C*_p_ = *C*_12_ − *C*_44_

11
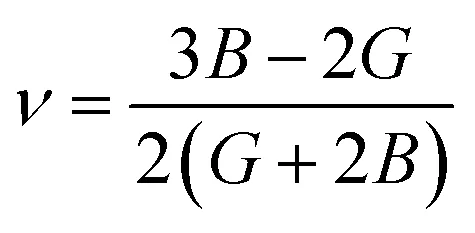
12
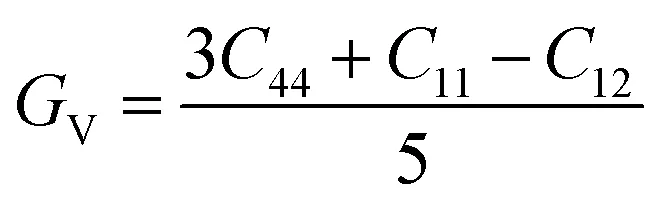
13
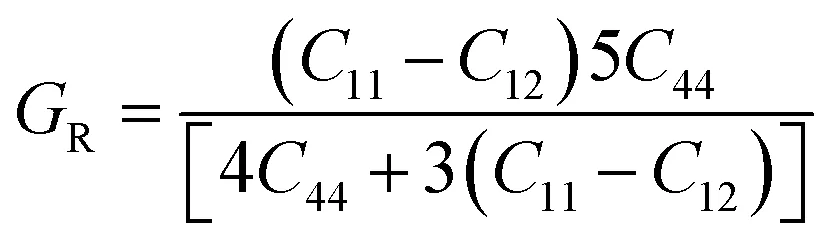


The *G* is calculated by using Navier's equation:^53^14
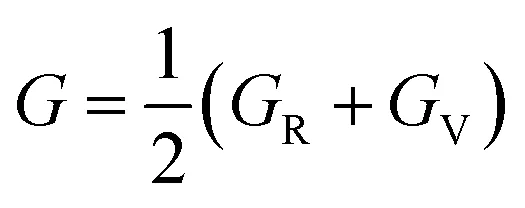


Typically, the *B* represents the resistance of a system to compressive strain, while *E* describes the inherent stiffness of a material and *G* characterizes its resistance to shape deformation and is often correlated with its hardness. Compared to Rb_2_InAsF_6_ (38.36) and Cs_2_InAsF_6_ (40.10), K_2_InAsF_6_ has a larger *B* value (42.06), reflecting that it is more resistive to both compounds. In contrast, *E* is larger for Rb_2_InAsF_6_ (50.18) than K_2_InAsF_6_ (45.41) and Cs_2_InAsF_6_ (39.78), indicating its higher stiffness than the other two. Similarly, the computed *G* for Rb_2_InAsF_6_ (19.09) is higher than K_2_InAsF_6_ (17.20) and Cs_2_InAsF_6_ (14.99). Consequently, Rb_2_InAsF_6_ exhibits greater resistance to shape distortion and hardness than K_2_InAsF_6_ and Cs_2_InAsF_6_.

To further assess the ductility of studied materials some key factors including Pugh's ratio (*B*/*G*), *C*_p_ and *ν* are calculated. The *B*/*G* ratio >1.75, positive value of *C*_p_ and *ν* > 0.26 indicate their ductile character and smaller quantities than these limits suggests their brittle nature. Notably, the calculated Pugh's ratio >1.75 and positive *C*_p_ values confirm their ductile nature, further supported by computed *ν* values of 0.32, 0.314 and 0.327 for K_2_InAsF_6_, Rb_2_InAsF_6_ and Cs_2_InAsF_6_, respectively.

The Zener anisotropy (*A*_z_) serves as an important indicator, as it characterizes the directional dependence of physical characteristics of a solid under uniform mechanical stress. *A* value of *A* = 1, corresponds to ideal isotropic behavior, whereas *A* ≠ 1 reflects elastic anisotropy and extent of deviation from isotropic behavior. The values of *A*_z_ as shown in Table 2, show significant deviation from unity, confirm their elastically anisotropic nature. The melting temperature (*T*_m_) evaluates the thermal stability of materials and their applicability in device fabrication. The *T*_m_ is estimated using the calculated value of *C*_11_ through the following expression:^54^

15 *T*_m_ = [553 + *C*_11_(5.9)] ± 300

The values of *T*_m_ for K_2_InAsF_6_, Rb_2_InAsF_6_, and Cs_2_InAsF_6_ are 1014 K, 964 K, and 1026 K, respectively, indicating the stability of studied compounds up to these elevated temperature values.

To evaluate the direction-dependent behavior of elastic parameters, the three-dimensional (3D) contour plots and associated two-dimensional (2D) planar projections were constructed using ELATE code.^55^ As observed in Fig. (2–4), the parameters *E* and *G* exhibit substantial anisotropy, as their 3D and 2D plots deviate from ideal spherical and circular geometries, respectively. The *ν* also demonstrates anisotropic characteristics, with regions of relatively high and low values, depending on orientation, reflecting directional variation in the transverse strain response under applied stress. This behavior indicates non-uniform mechanical coupling within the crystal lattice. In contrast, linear compressibility (*β*) shows nearly isotropic behavior as depicted by its almost spherical 3D surfaces and circular 2D projections. To quantitatively validate the anisotropy, the maximum and minimum values and corresponding anisotropy ratios of *E*, *G*, *ν* and linear compressibility (*β*) are calculated and presented in Table 3. The linear compressibility exhibits an isotropic behavior, as confirmed by anisotropy ratio *A* = 1. In contrast, all other parameters show a significant level of anisotropy as indicated by their non-unity *A* ratios. In particular, Cs_2_InAsF_6_ exhibits a comparatively higher degree of anisotropy in *E*(≈1.93) and *ν*(≈3.83), whereas K_2_InAsF_6_ and Cs_2_InAsF_6_ show slightly stronger anisotropy in *G* compared to Rb_2_InAsF_6_. Structural stability is a prerequisite for the materials used for the PV and catalytic applications. In addition, elastic anisotropy and effective mechanical response are some of the most important characteristics used to examined their long-term performance for TE and optoelectronic devices. The studied materials are ductile and can resist deformation without undergoing any fracture. The calculated anisotropy indicates that elastic properties are direction-dependent, which may influence the distribution of stress and strain under external compressive loading. These characteristics render their potential for flexible optoelectronic and sensor applications, where materials may experience loading, bending and stretching during their operation. Overall, the attractive elastic behavior with significant elastic anisotropy provides important insights into their practical implementation in advanced technological usages.

**Fig. 2:**
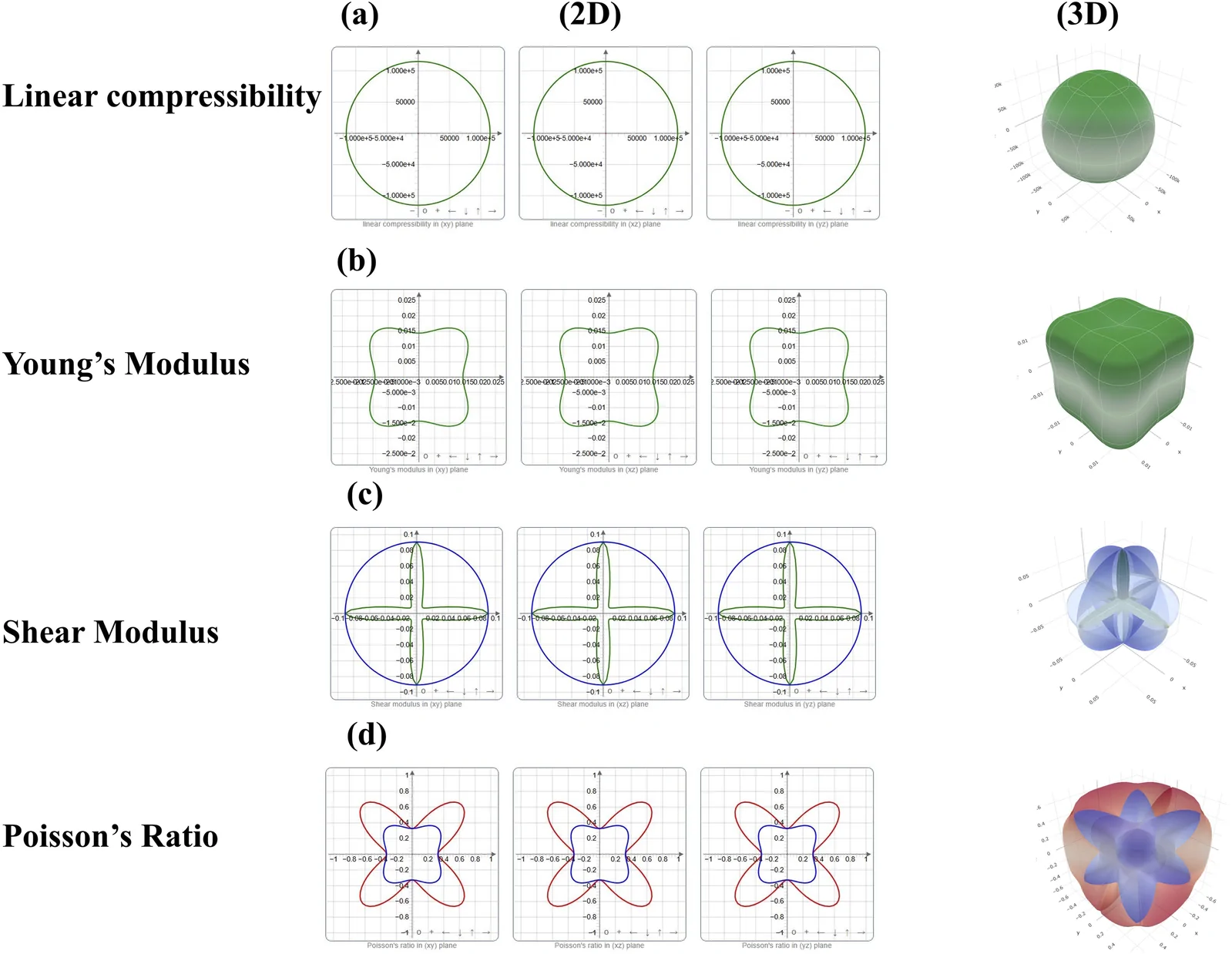
2D and 3D directional plots of (a) linear compressibility, (b) *E*, (c) *G*, and, (d) *ν* of K_2_InAsF_6_.

**Fig. 3:**
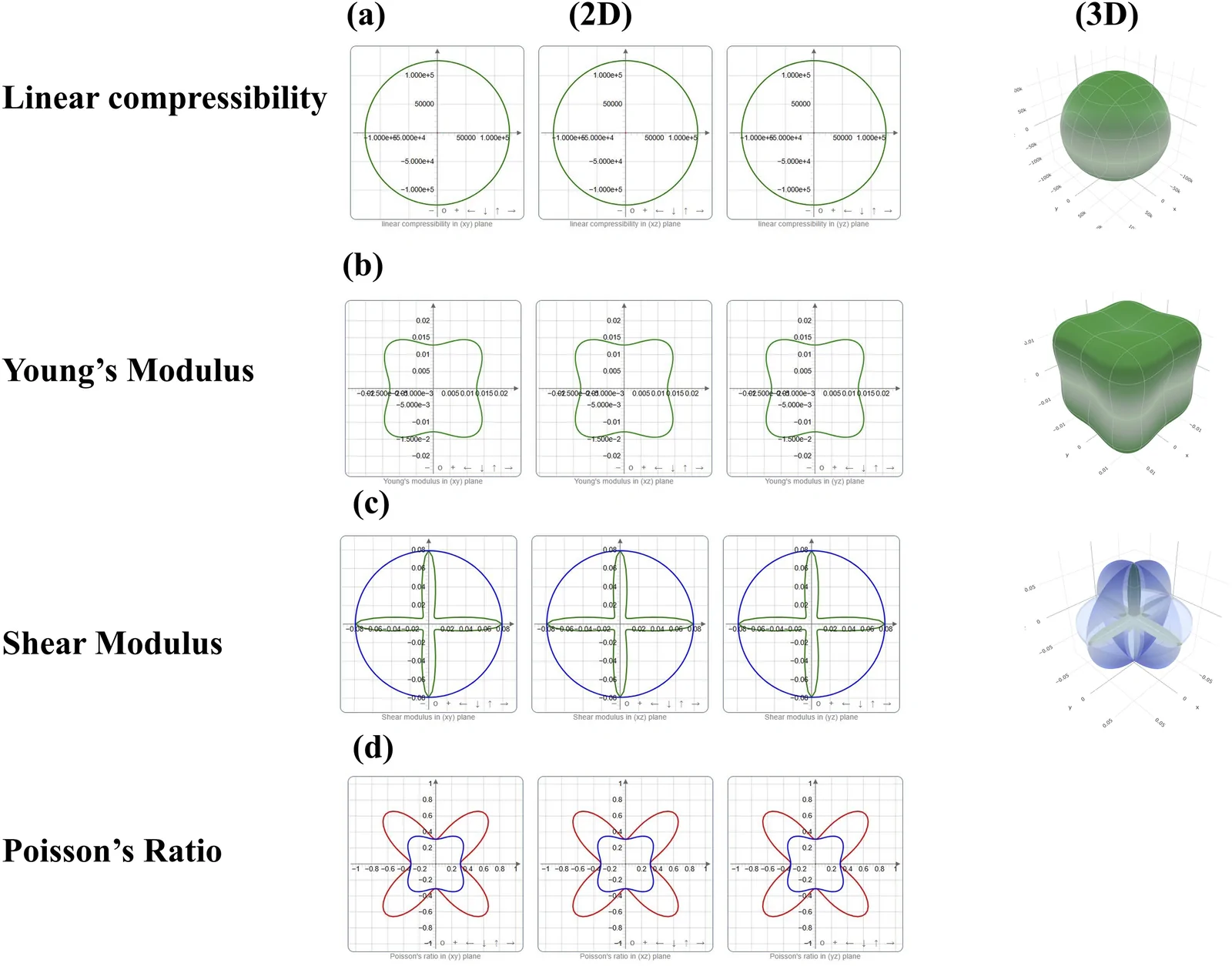
2D and 3D directional plots of (a) linear compressibility, (b) *E*, (c) *G*, and, (d) *ν* of Rb_2_InAsF_6_.

**Fig. 4:**
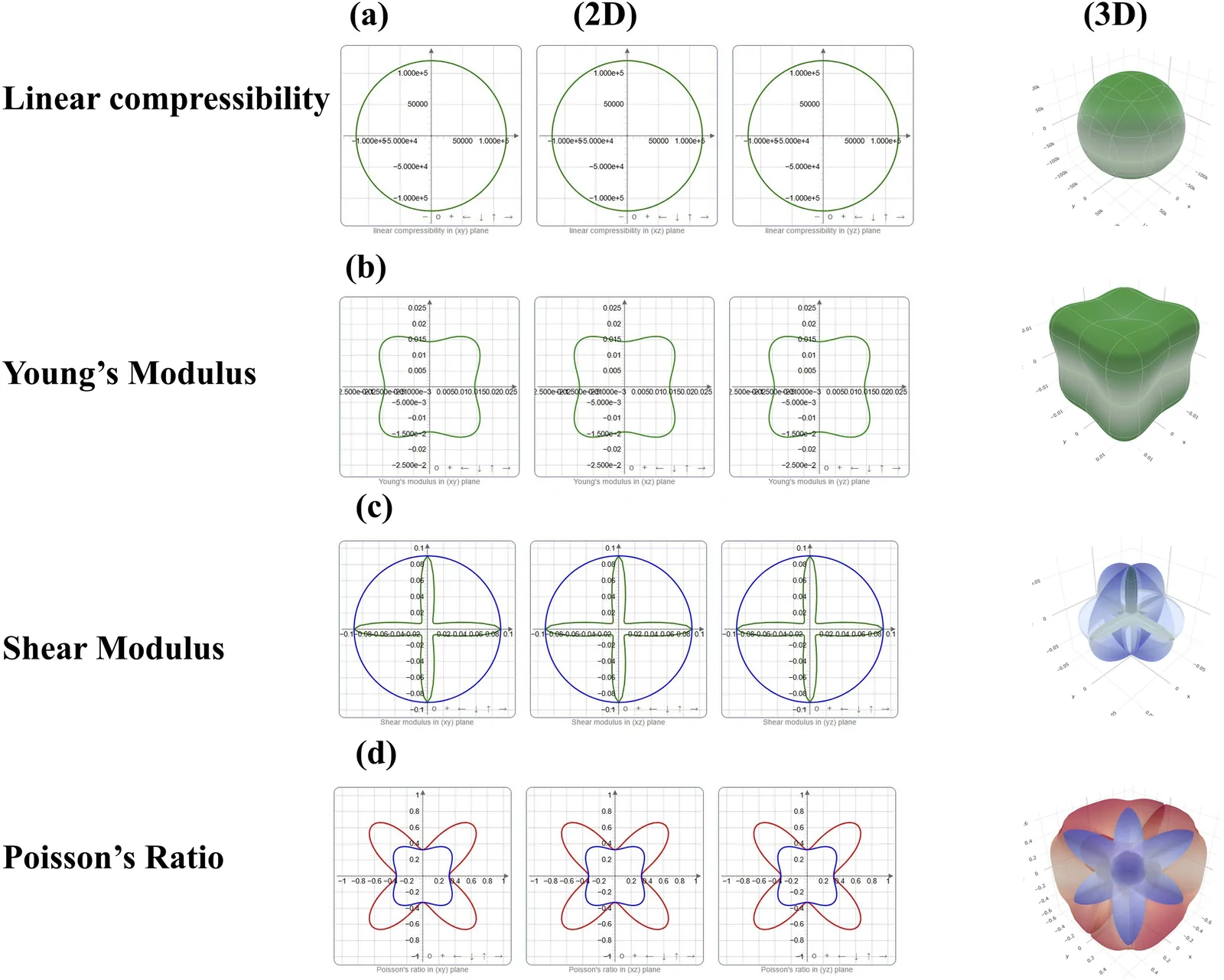
2D and 3D directional plots of (a) linear compressibility, (b) *E*, (c) *G*, and, *ν* of Cs_2_InAsF_6_.

**Table 3:** The minimum and maximum values of the elastic moduli, along with their corresponding anisotropy ratios, for K_2_InAsF_6_, Rb_2_InAsF_6_ and Cs_2_InAsF_6_

Compounds	*β* (GPa^−1^)			*E* (GPa)			*G* (GPa)			*ν*		
	*β*_min_	*β*_max_	*A*	*E*_max_	*E*_min_	*A*	*G*_max_	*G*_min_	*A*	*ν*_max_	*ν*_min_	*A*
K_2_InAsF_6_	0.0079	0.0079	1	63.6	46.0	1.38	26.92	12.65	2.13	0.431	0.17	2.53
Rb_2_InAsF_6_	0.0083	0.0083	1	71.8	40.02	1.79	29.89	15.08	1.98	0.201	0.114	1.76
Cs_2_InAsF_6_	0.0086	0.0086	1	58.32	30.21	1.93	23.39	11.04	2.12	0.556	0.145	3.83

### 3.3 Electronic properties

The TE response of a system is strongly dependent on its electronic structure. Generally, materials with flat bands near Fermi level and moderate band gaps are considered as potential TE materials, as they can provide high density of states and favorable transport characteristics.^56^

The electronic properties of DHPs X_2_InAsF_6_ (X = K, Rb, Cs) including band structures (BSs), total and projected density of states (DOS) were analyzed through the mBJ potential. The computed BSs of X_2_InAsF_6_ are presented in Fig. (5–7) along the high symmetry *k*-point path (W–L–Γ–X–W–K) of irreducible BZ. The BS analysis confirms that studied materials are direct *E*_g_ semiconductors, with the valence band maxima (VBM) and conduction band minima (CBM) positioned at Γ point, indicating favourable electronic transition. The computed *E*_g_ values are 2.61, 2.70, and 2.74 eV for K_2_InAsF_6_, Rb_2_InAsF_6_, and Cs_2_InAsF_6_, correspondingly. It is noted that band gap increases by substituting the A-site cations from K to Rb and Rb to Cs. This enhancement is mainly associated with variation in ionic size and atomic characteristics of A-site cations, which modify the crystal structure and electronic band dispersion. These findings show good correspondence with previous studies on DHPs, including X_2_CuInCl_6_ (X = K, Rb, and Cs) and X_2_GaAgF_6_ (X = Na/K/Rb, and Cs).^57,58^

**Fig. 5:**
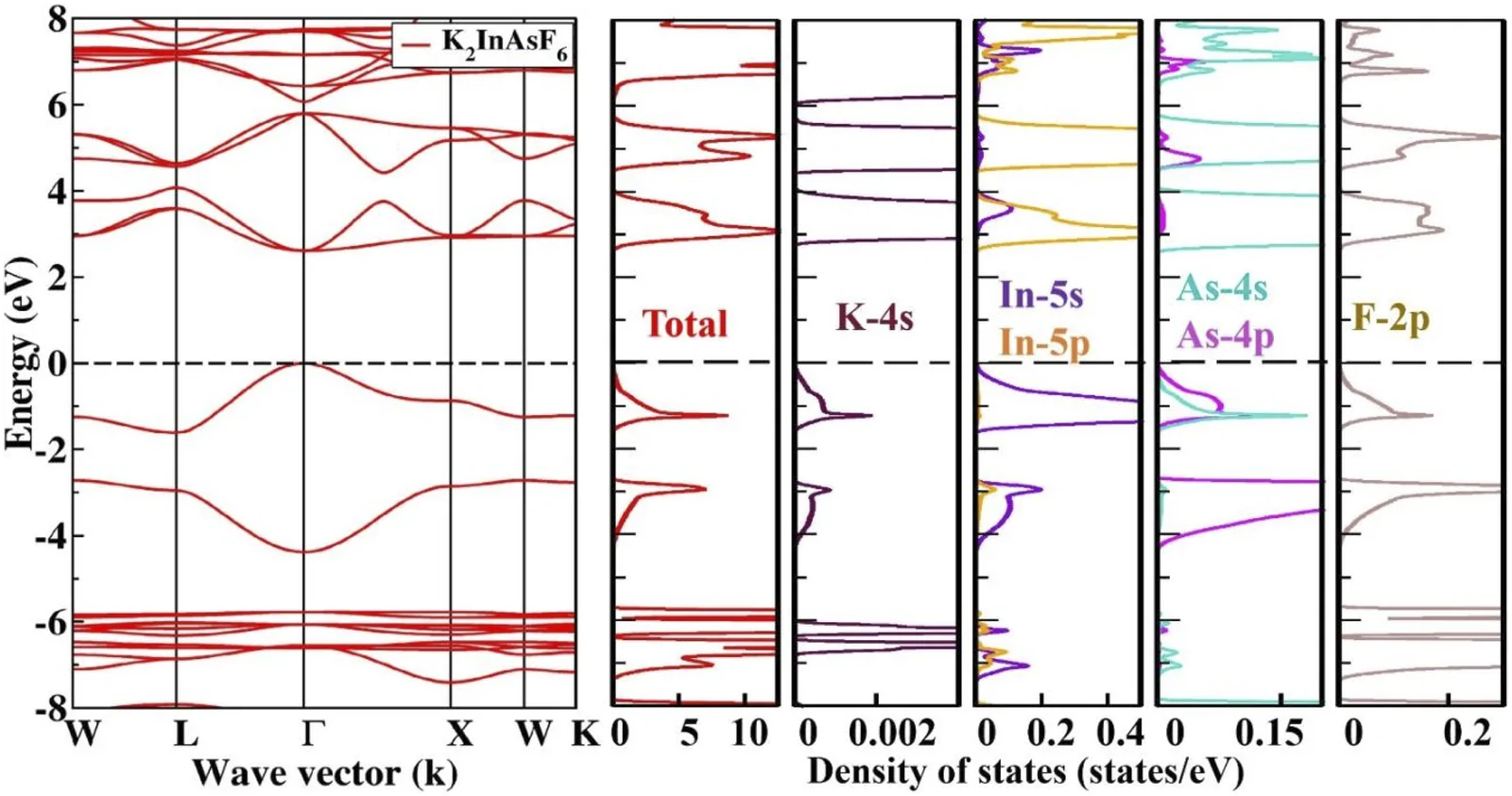
BS (left panel) and total, and partial DOS (right panel) for K_2_InAsF_6_.

**Fig. 6:**
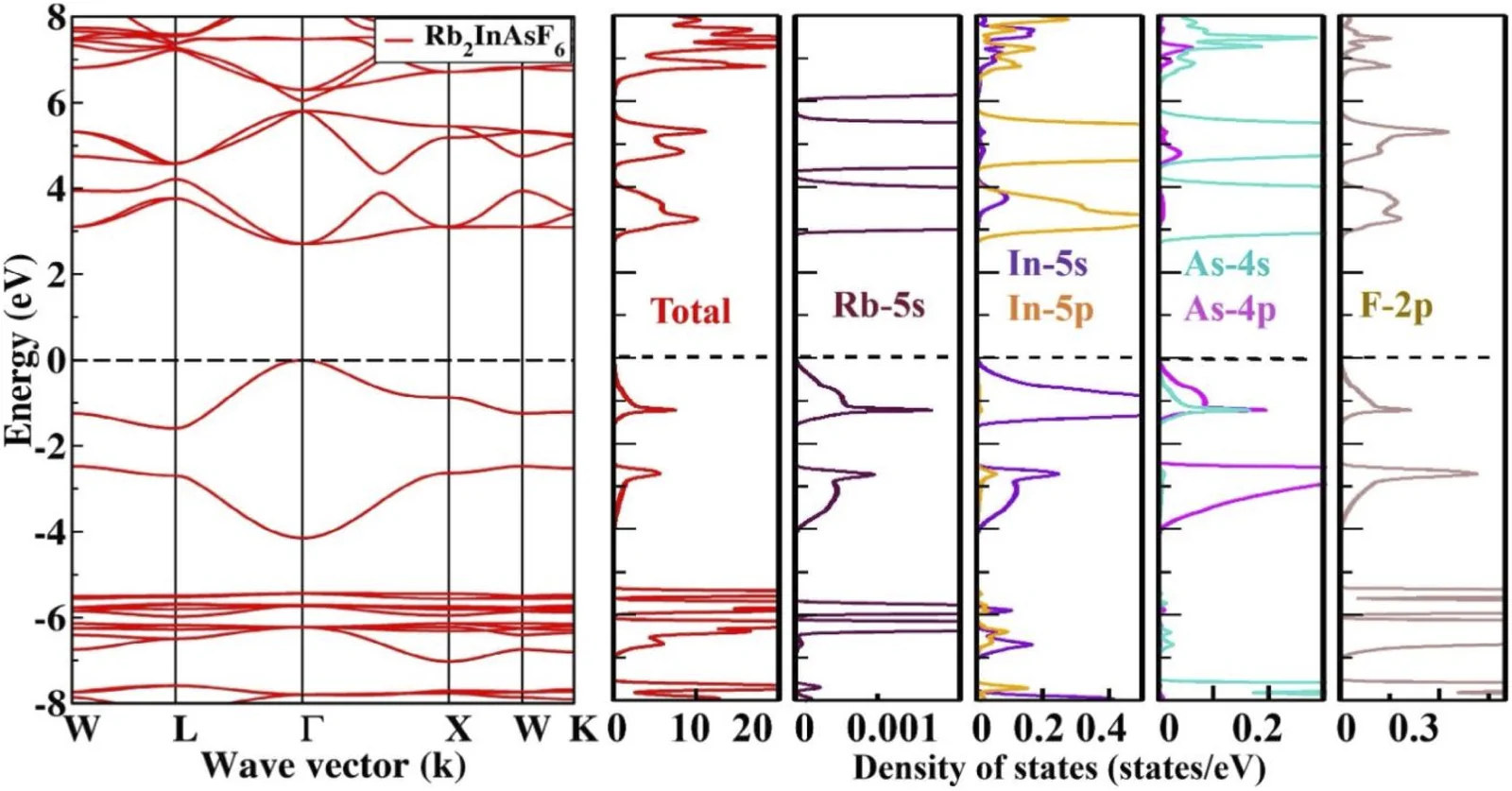
BS (left panel) and total, and partial DOS (right panel) for Rb_2_InAsF_6_.

**Fig. 7:**
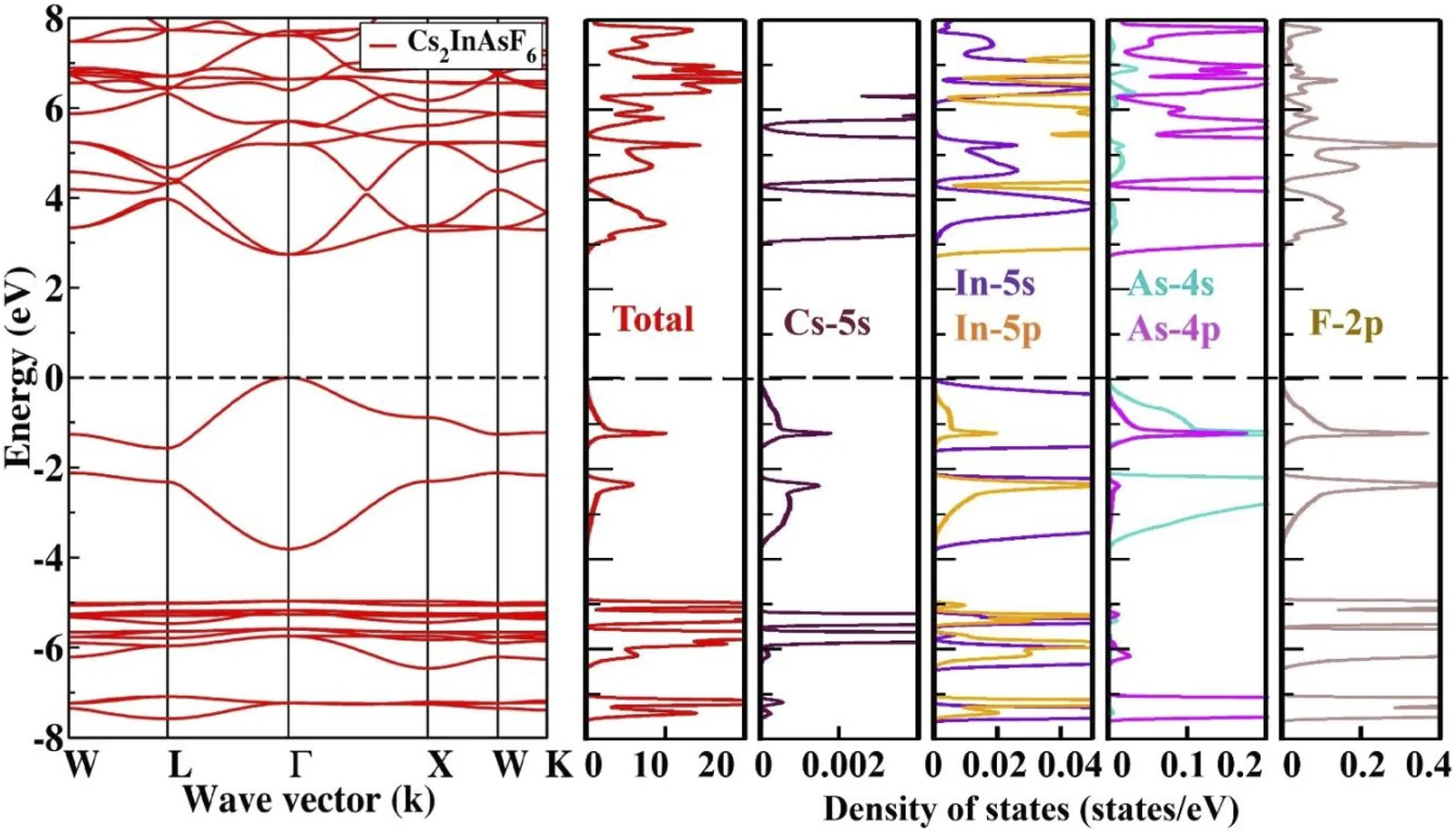
BS (left panel) and total, and partial DOS (right panel) for Cs_2_InAsF_6_.

The TDOS and PDOS graphs for X_2_InAsF_6_ DHPs are shown in Fig. (5–7).The TDOS provides knowledge on the distribution of total electronic states across different energy ranges, giving an overall picture of electronic structure, whereas PDOS describes the contributions of the individual orbitals of constituent elements, enabling an in-depth understanding of orbital-resolved electronic states and their role in formation of VB and CB. The PDOS graphs reveal that electronic structure of all three compounds exhibit similar characteristics. The VB is primarily composed of In-5s, As-4p/4s, F-2p and alkali–metal s states (K-4s, Rb-5s and Cs-5s), while CB is majorly consisted of In-5p, and As-4s/4p states, with additional contributions from F-2p. Strong orbital hybridization is observed in both VB (0 to −4 eV and −6 to −8 eV) and CB (3–8 eV), indicating significant electronic interactions between constituent atoms. Overall, replacing the K with Rb or Cs has strong influence on the *E*_g_ values and electronic BS of studied DHPs (Table 4).

**Table 4:** Band gaps and optical parameters for K_2_InAsF_6_, Rb_2_InAsF_6_ and Cs_2_InAsF_6_

Materials	Bandgap (eV)	*ε*_1_(0)	*n*(0)	*R*(0)
K_2_InAsF_6_	2.61	2.91	1.74	0.07
Rb_2_InAsF_6_	2.70	2.91	1.73	0.07
Cs_2_InAsF_6_	2.74	3.09	1.74	0.07

### 3.4 Optical properties

To further elucidate the optical response of the probed DHPs, a thorough examination of their optical parameters was performed. Optical parameters provide valuable information regarding optical transitions between the VB and the CB, as well as the interaction of electromagnetic (EM) radiation with material's electronic BS.^59^ Since the studied compounds exhibit direct band gaps, efficient interband transitions can occur without the assistance of phonons, thereby minimizing the energy losses caused by lattice vibrations.^60^ Because the optical character of a compound is frequency dependent, the complex dielectric constant *ε*(*ω*) consisting of the real *ε*_1_(*ω*) and imaginary components *ε*_2_(*ω*)^61^ was used to evaluate the optical features of X_2_InAsF_6_ compounds.

16 *ε*(*ω*) = *ε*_1_(*ω*) + *iε*_2_(*ω*)

17
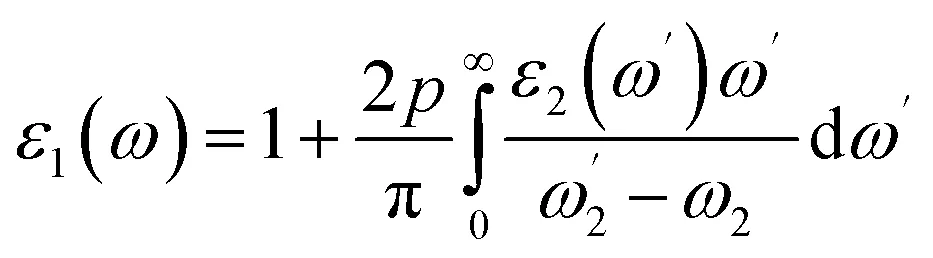


The *ε*_1_(*ω*) illustrates the optical polarizability of a material under an applied EM field and reflects its energy storage capacity. The calculated *ε*_1_(0), describes the low-energy dielectric behavior of a material. The calculated *ε*_1_(0) are 2.9 for K_2_InAsF_6_ and Rb_2_InAsF_6_ and 3.0 for Cs_2_InAsF_6_ (see Fig. 8(a)). These findings show good correspondence with the Penn relation, which links the *E*_g_ to the *ε*(*ω*) through the following expression:^62^18
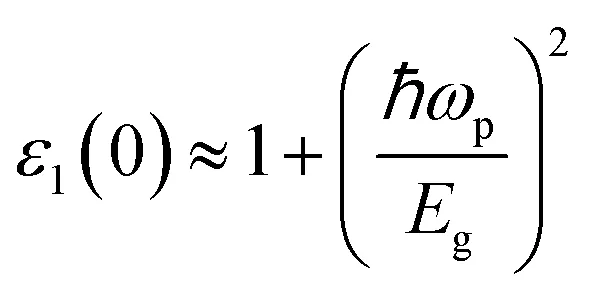


**Fig. 8:**
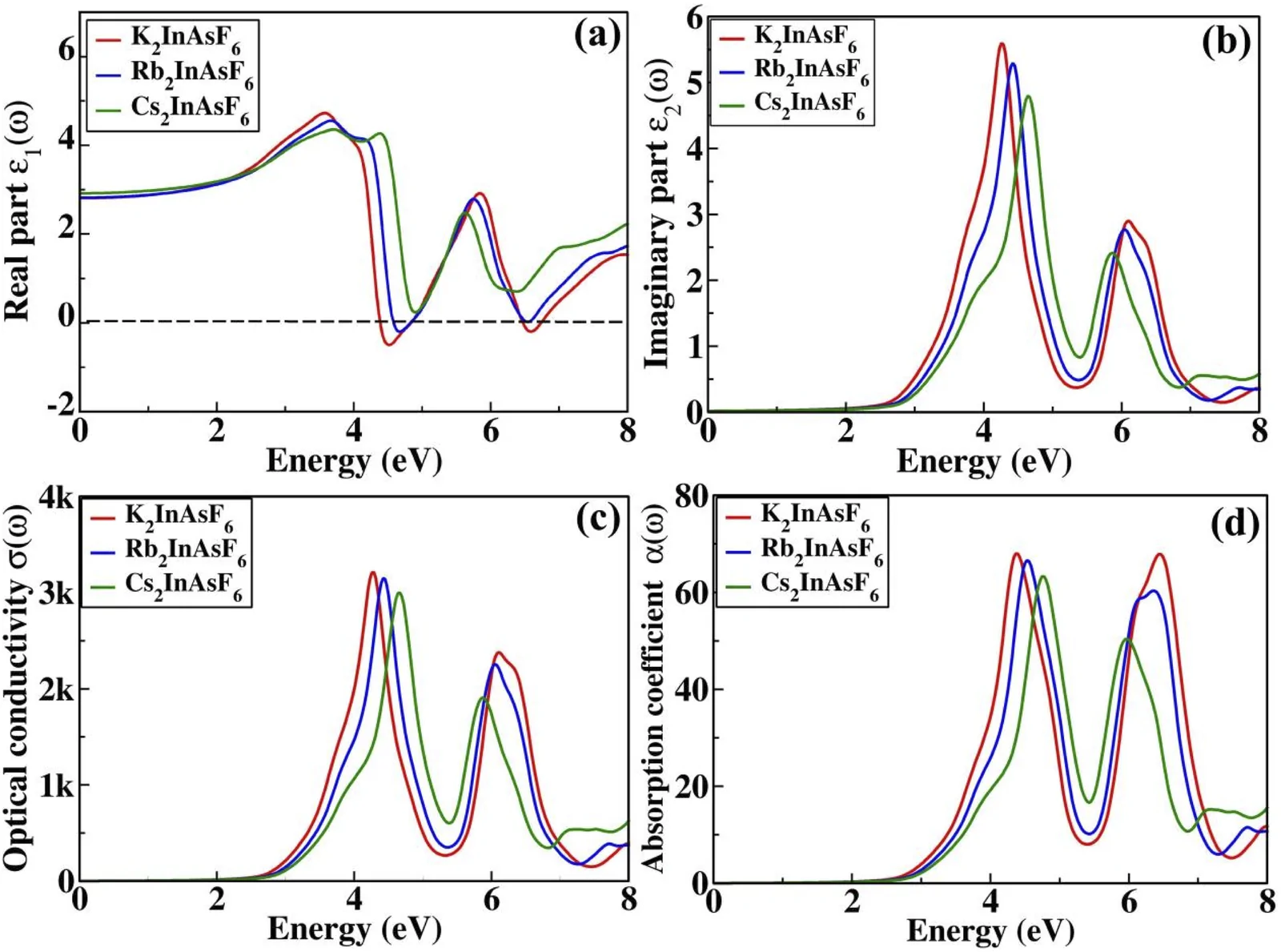
(a) *ε*_1_(*ω*), and (b) *ε*_2_(*ω*), (c) *σ*(*ω*), (d) *α*(*ω*).

According to this relation, materials with larger band gaps exhibit lower *ε*_1_(0) values due to reduced optical polarizability. The low *ε*_1_(0) values of the studied compounds are comparable with their wide *E*_g_ semiconducting character. The *ε*_1_(*ω*) rises gradually with rising photon energy and attains its maximum values within 3.5–4.0 eV. The extreme values are about 4.7 (K_2_InAsF_6_), 4.5 (Rb_2_InAsF_6_), and 4.4 (Cs_2_InAsF_6_), reflecting enhanced electronic polarization due to interband transitions. After this range, *ε*_1_(*ω*) spectra exhibits a sharp decline crossing the zero line near 4.3–4.7 eV. The *ε*_1_(*ω*) values indicate the metallic-like response of materials in this energy range, where the material strongly reflects the incident EM waves. Subsequently, *ε*_1_(*ω*) rises again and attains secondary maxima around 5.6–5.9 eV, followed by another zero crossing near 6.4–6.8 eV. Within higher energy regions, *ε*_1_(*ω*) increases steadily and regains positive values. Among all three compounds, Cs_2_InAsF_6_ exhibits broad spectral variation, whereas K_2_InAsF_6_ exhibits a strong dielectric response, suggesting a subtle difference in their electronic structure and optical polarization behavior.

The *ε*_2_(*ω*) illustrates the absorptive behavior of a material to incident EM radiation, thereby providing direct insight into interband electronic transitions:^63^19
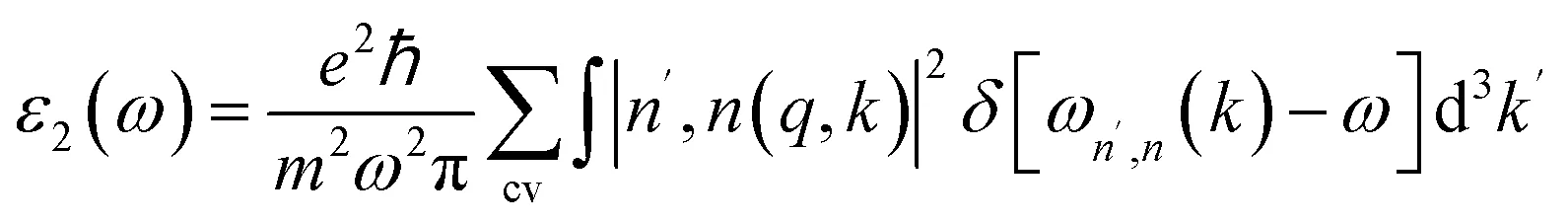
Here, *m*, *q*, and *ω* denote the electron mass, charge, and the angular frequency, respectively. *ε*_2_(*ω*) is strongly governed by the BS of a material (see Fig. 8(b)). For energies below the fundamental band gaps, *ε*_2_(*ω*) remains negligibly small, indicating the absence of optical absorption. Accordingly, the absorption threshold appears at 2.60, 2.72, and 2.76 eV for K_2_InAsF_6_, Rb_2_InAsF_6_, and Cs_2_InAsF_6_, respectively, corresponding to their calculated band gap energies. The first prominent peaks of *ε*_2_(*ω*) with maximum values of 5.6, 5.2, and 4.9 occur at the photon energies of 4.2, 4.4, and 4.6 eV for K_2_InAsF_6_, Rb_2_InAsF_6_, and Cs_2_InAsF_6_, respectively. A second set of pronounced peaks appears within 5.8–6.2 eV range for all three compounds, indicating the occurrence of additional interband transitions at higher energies. The high-intensity peaks present in the ultraviolet region (UV) demonstrates the interaction of investigated materials with high energy photons. These features highlight their potential for optoelectronic usages like photodetectors and other optical technologies requiring efficient absorption of high-energy photons.

The *α*(*ω*) as well as optical conductivity *σ*(*ω*) were explored to further assess their absorption characteristics. The absorption coefficient represents light absorption inside the material, while optical conductivity demonstrates the generation and transportation of charge carriers induced by photon absorption.^64^ The onset of *σ*(*ω*) occurs at 4.2, 4.4, and 4.7 eV for K_2_InAsF_6_, Rb_2_InAsF_6_, and Cs_2_InAsF_6_, respectively, with corresponding peak values of 3.2 × 10^3^, 3.1 × 10^3^, and 3.0 × 10^3^ Ω^−1^ cm^−1^ as shown in Fig. 8(c). Additional conductivity peaks were observed in the 5.8–6.1 eV range with reduced magnitudes of 2.3× 10^3^, 2.2 × 10^3^, and 1.9 × 10^3^ Ω^−1^ cm^−1^. The existence of these curves in the UV span indicates good efficiency of carrier generation under UV irradiation. Below the *E*_g_ energies, the *α*(*ω*) spectrum does not show any absorption and possess transparent character, as depicted in Fig. 8(d). With increment in energy, the *α*(*ω*) also elevates and reaches its peak value at 4.3, 4.5, and 4.7 eV for K_2_InAsF_6_, Rb_2_InAsF_6_, and Cs_2_InAsF_6_, respectively. Secondary prominent peaks appear between 5.9 and 6.4 eV with magnitudes of 68× 10^4^, 60 × 10^4^, and 50 × 10^4^ cm^−1^. The significant *α*(*ω*) and *σ*(*ω*) peaks appearing in the UV range describe the capability of these compounds for UV optoelectronic and photo detection systems.

The refractive index *n*(*ω*) reflects the propagation of EM radiation through a substance and provides information about the transparency and light–matter interaction (see Fig. 9(a)).^65^ All compounds show a static refractive index of 1.7, suggesting similar polarization at low frequencies. The *n*(*ω*) spectra closely mirrors of *ε*_1_(*ω*) because they are intrinsically linked. The *n*(*ω*) is highest at 3.5 eV with a value of 2.28 for both K_2_InAsF_6_ and Rb_2_InAsF_6_ and is slightly lower for Cs_2_InAsF_6_ with a value of 2.21 at 4.42 eV. After these energy ranges, the *n*(*ω*) falls slowly and reaches another peak at around 5.8 eV with a value of 1.7 for all the examined compounds. The extinction or attenuation coefficient *k*(*ω*) is directly related to *ε*_2_(*ω*) and it is a measure of the attenuation of EM radiation within a material. In Fig. 9(b), the highest *k*(*ω*) curves are noted for K_2_InAsF_6_ at 4.3, for Rb_2_InAsF_6_ at 4.5 eV, and for Cs_2_InAsF_6_ at 4.7 eV. The observed low *k*(*ω*) values in the low-energy region indicate weak optical losses and good transparency, while enhanced attenuation occurring at higher photon energies are indication of increased interband transitions. Furthermore, the *k*(*ω*) spectra exhibit spectral features similar to those of *σ*(*ω*) and *ε*_2_(*ω*), reflecting their common origin in optical absorption processes.

**Fig. 9:**
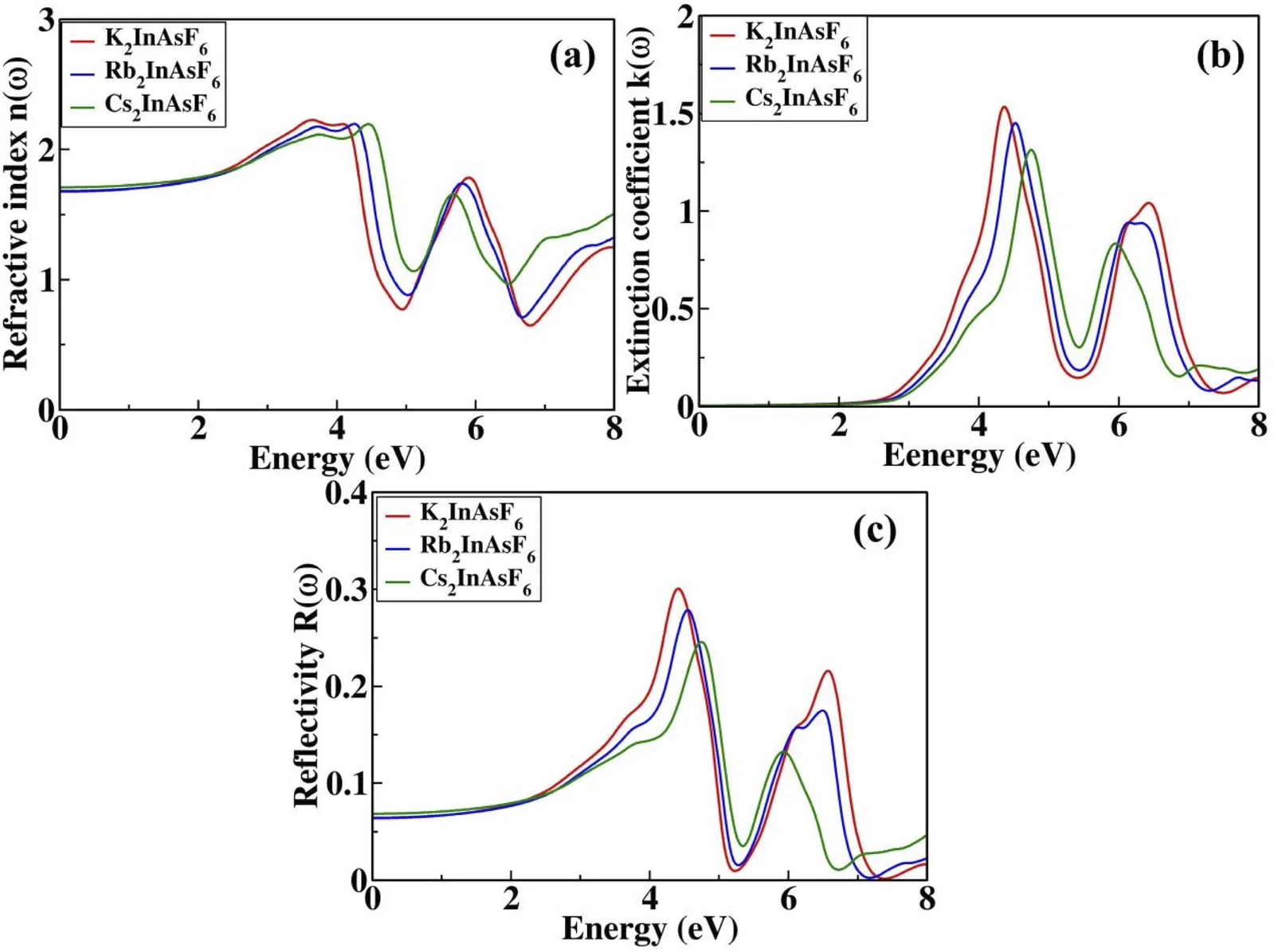
(a) *n*(*ω*), (b) *k*(*ω*), and (c) *R*(*ω*).

Lastly, the optical reflectivity *R*(*ω*) was determined that provides information about the portion of falling EM radiation reflected by the substance's exterior. The computed static reflectivity is 0.07 for all three compounds; exhibiting negligible reflection losses in the low-energy regime (see Fig. 9(c)). The *R*(*ω*) spectra rises steadily with photon energy and reaches extreme values of 0.30/0.27 for K_2_InAsF_6_/Cs_2_InAsF_6_ compounds, correspondingly. The low reflectivity in the vicinity of band gap region signifies the high transparency of investigated DHPs. The strong *α*(*ω*) combined with low reflectance in the UV region signify that studied DHPs are suitable for UV-shielding and optical coatings.

### 3.5 Thermoelectric properties

Among sustainable energy technologies, TE materials provide a promising route by directly harvesting heat into usable heat. TE materials offer several advantages including environmental friendly, high reliability and compatibility with renewable energy systems.^66^ Their performance is quantified through figure of merit (*ZT*), which requires an optimal balance among Seebeck coefficient (*S*), thermal conductivity (*κ*/*τ*), and electrical conductivity (*σ*/*τ*).^67^ Thermoelectric features of X_2_InAsF_6_ (X = K/Rb/Cs) were determined to assess their suitability for power generation usages within 300–800 K.

The *σ*/*τ* spectra is shown in Fig. 10(a) and represents the ability for charge transport within a material, the ease with which charge carriers move through the crystalline solid in an external electric field.^68^ The *σ*/*τ* grows consistently with temperature, attaining extreme values of 0.158, 0.154 and 0.163 × 1019 (Ω cm s)^−1^ for K_2_InAsF_6_, Rb_2_InAsF_6_, and Cs_2_InAsF_6_, respectively at 800 K. The Cs_2_InAsF_6_ exhibit a slightly higher *σ*/*τ* than K_2_InAsF_6_ and Rb_2_InAsF_6_, which shows the lower resistivity and the efficient charge transport than K_2_InAsF_6_ and Rb_2_InAsF_6_. This higher value of *σ*/*τ* at higher temperature is ascribed to improved carrier transport, which is ascribed to thermal activation of carriers at high temperature. Fig. 10(b) represents the thermal conductivity (*κ*/*τ*) of studied compounds, which characterizes their capacity to pass heat. It measures the transportation rate of thermal energy into a substance and provides valuable insight into mechanisms governing heat transfer.^69^ Thermal conductivity reflects the combined contributions of lattice vibrations (*κ*_l_) and charge carrier (*κ*_e_) to overall thermal transport, thereby serving as key terms for analyzing overall TE response of a system. The *κ*_l_ is assessed by employing Slack's calculation.^70^ The *κ*_l_ is found to exhibit decreasing trend with temperature and its contribution becomes less significant at temperatures above room temperature (Fig. 1S). In contrast, *κ*_e_/*τ* is computed using the BoltzTrap code and exhibits a significant influence on TE performance of a material. The *κ*_e_/*τ* at 300 K is calculated to be 0.055 × 10^14^ W/K ms for all studied compounds as presented in Fig. 10(b). The *κ*_e_/*τ* rises linearly with temperature, reaching peak values of 0.055 × 10^14^ for K_2_InAsF_6_/Rb_2_InAsF_6_ and 1.01 × 10^14^ W/Kms for Cs_2_InAsF_6_ at 800 K. The Cs_2_InAsF_6_ exhibit a higher *κ*_e_/*τ* value than that of K_2_InAsF_6_ and Rb_2_InAsF_6_.

**Fig. 10:**
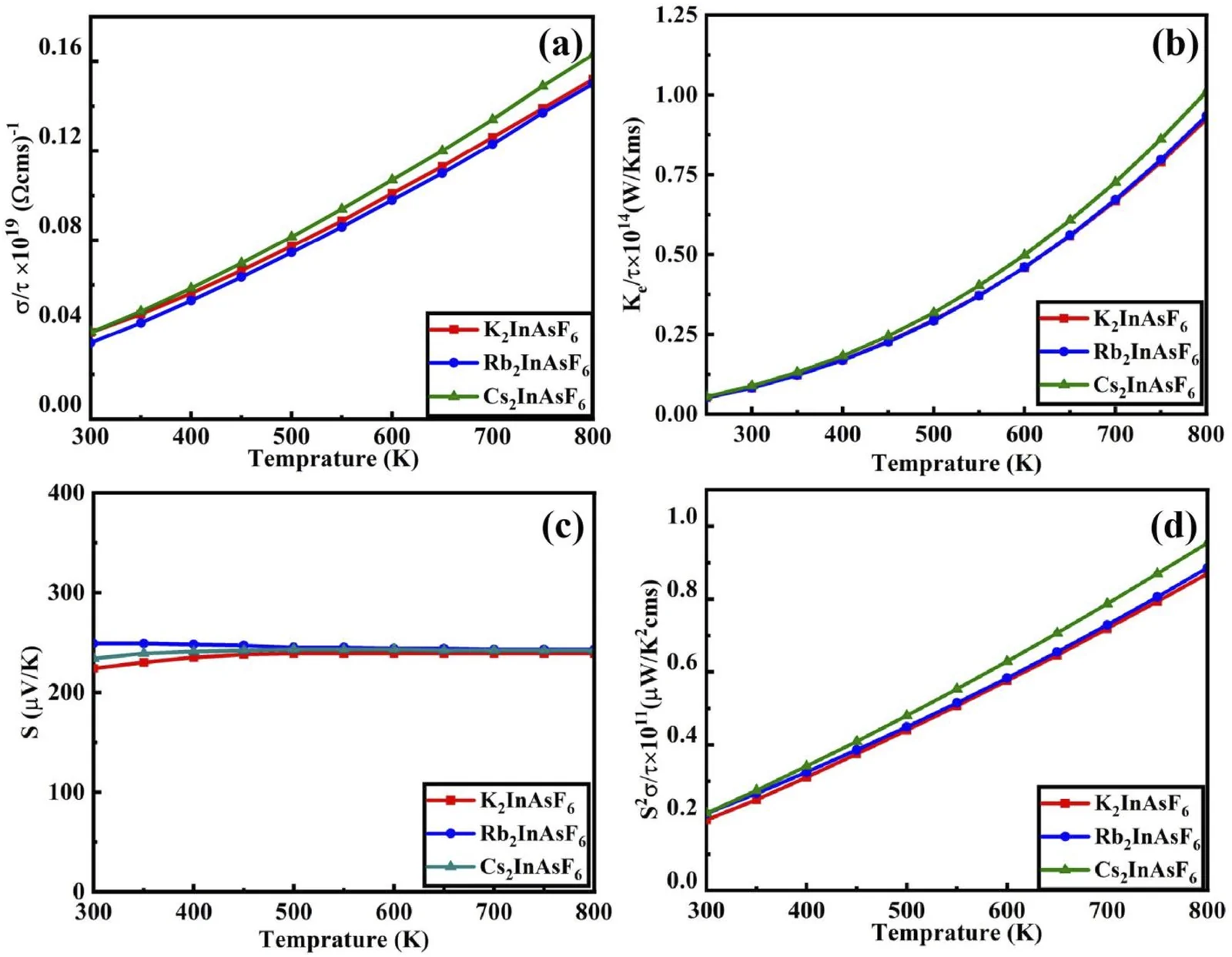
(a) *σ*/*τ*, (b) *K*_e_/*τ*, (c) *S*, and (d) PF.

The *S* is a key indicator that quantifies the voltage developed within substance by the application of temperature gradient. The polarity of *S* describes the type of primary charge carriers: with negative and positive signs suggesting hole-dominated and electron-dominated conduction, respectively.^71^ The behavior of *S* can be interpreted through Pisarenko relation, which establishes a relationship between effective mass and carrier concentration:^72^20
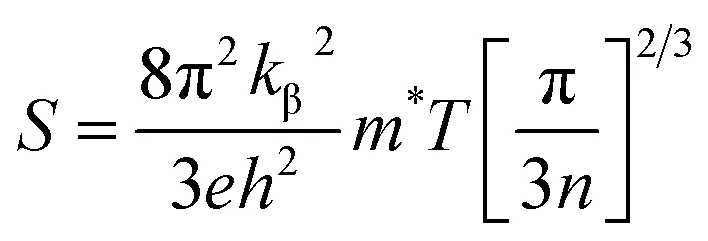


For efficient thermoelectric performance, a relatively high Seebeck coefficient is desirable, with values exceeding 200 µV K^−1^ typically considered favourable. The S is determined to be 224, 234 and 249 µV K^−1^ for K_2_InAsF_6_, Rb_2_InAsF_6_, and Cs_2_InAsF_6_, respectively (see Fig. 10(c)). For K_2_InAsF_6_ and Cs_2_InAsF_6_ the *S* exhibits a slight increase with temperature and becomes nearly temperature-independent above 450 K. In contrast, for Rb_2_InAsF_6_ the Seebeck coefficient decreases marginally from 249 at 300 to 242 µV K^−1^ at 800 K. The *S* remains significantly high throughout the investigated temperature range, reflecting the aptness of proved DHPs for TE usages. The positive *S* values for studied compounds reflect their p-type semiconductor behavior.

The power factor (PF), defined as PF = *S*^2^*σ*, reflects the material's capability to produce electrical output from a given temperature gradient while maintaining efficient charge transport.^73^ At 300 K, the calculated PF value for K_2_InAsF_6_ is 0.19 × 10^11^, whereas, Rb_2_InAsF_6_ and Cs_2_InAsF_6_ exhibit slightly higher values of 0.21 × 10^11^ µW K^2^ cm s^−1^ as observed in Fig. 10(d). For all three compounds, PF increases monotonically, reaching peak values of 0.87 × 10^11^, 0.88 × 10^11^, and 0.95 × 10^11^ µW K^2^ cm s^−1^ for K_2_InAsF_6_, Rb_2_InAsF_6_, and Cs_2_InAsF_6_, respectively at 800 K (see Table 5).

**Table 5:** TE parameter values for K_2_InAsF_6_, Rb_2_InAsF_6_ and Cs_2_InAsF_6_

Thermoelectric parameters	K_2_InAsF_6_		Rb_2_InAsF_6_		Cs_2_InAsF_6_	
	300 K	800 K	300 K	800 K	300 K	800K
*S*	224	239	249	243	234	242
(*κ*_e_/*τ*)	0.055	0.934	0.055	0.934	0.055	1.01
(*σ*/*τ*)	0.038	0.158	0.034	0.154	0.038	0.163
*ZT*	0.708	0.752	0.778	0.758	0.723	0.759
(*S*^2^*σ*/*τ*)	0.194	0.871	0.212	0.886	0.212	0.954

The TE efficacy of a material is quantified through *ZT*, which is represented as:^74^21
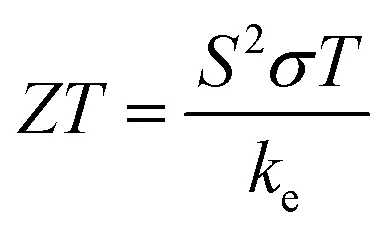


As observed in Fig. 11, the calculated *ZT* values are 0.70 for K_2_InAsF_6_ and 0.72 for Cs_2_InAsF_6_ at 300 K. The *ZT* value gradually rises with temperature, attaining a peak value of 0.75 at 800 K. In contrast, Rb_2_InAsF_6_ possesses slightly larger room-temperature *ZT* value of 0.77, which decreases slightly to 0.75 at 800 K. These *ZT* values are in good comparison to those for previously studied DHPs including Rb_2_AsAuBr_6_/Rb_2_AsAuCl_6_ (*ZT* ≈ 0.75), Cs_2_GaAgF_6_ (*ZT* ≈ 0.73), and Li_2_AgBiY_6_ (Y = Cl, Br, I; *ZT* ≈ 0.75).^75–77^

**Fig. 11:**
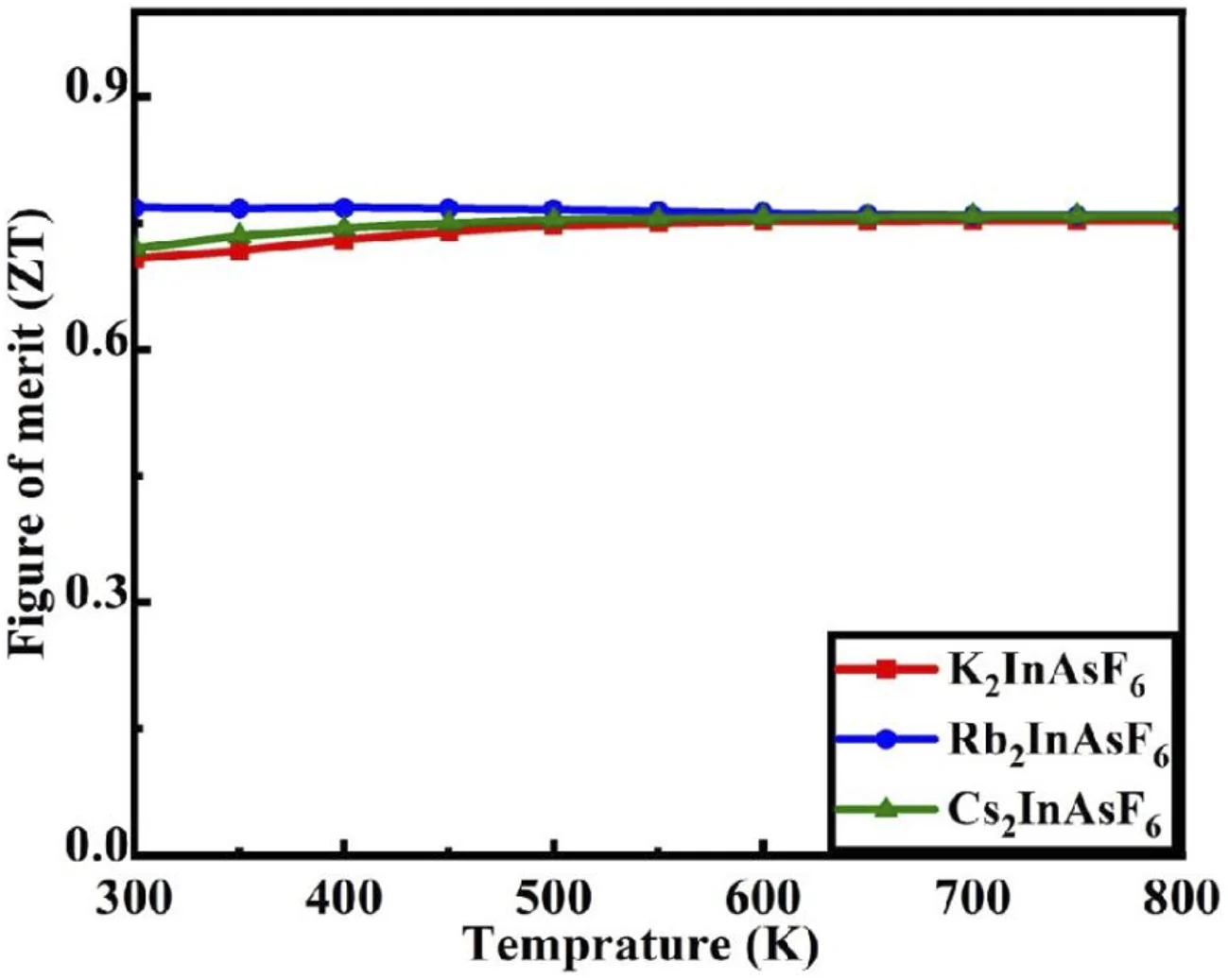
*ZT* of X_2_InAsF_6_ (X = K, Rb, Cs).

## 4. Conclusion

To summarize, different physical characteristics of DHPs X_2_InAsF_6_ (X = K/Rb/Cs) were thoroughly analyzed using first-principles simulations. In structural properties, the calculated negative formation enthalpy confirmed their thermodynamic stability. The computed Goldschmidt tolerance factors of 1 for K_2_InAsF_6_ and 0.99 for both Rb_2_InAsF_6_ and Cs_2_InAsF_6_, together with other stability parameters confirm their structural stability. The ground state lattice constants were computed to be 9.1561 Å for K_2_InAsF_6_, 9.2267 Å for Rb_2_InAsF_6_, and 9.3634 Å for Cs_2_InAsF_6_. The substitution of alkali metal cations from K to Rb and subsequently to Cs increases the *E*_g_ from 2.61 to 2.74 eV. The widened band gap results in a shift of the absorption edge from the visible to the UV region, suggesting their suitability for diverse optoelectronic devices. Their mechanical stability is verified by the compliance of the elastic parameters with the Born–Huang stability criteria, while surface plots reveal their elastic anisotropic behavior. Furthermore, thermoelectric calculations yield high power factors of 0.87, 0.88, and 0.95 × 10^11^ µW K^2^ cm s^−1^ at 800 K for K_2_InAsF_6_, Rb_2_InAsF_6_, and Cs_2_InAsF_6_, respectively, along with *ZT* values exceeding 0.7. These findings indicate that X_2_InAsF_6_ DHPs are favorable Pb-free materials for high temperature power generation applications.

## Conflicts of interest

There are no conflicts to declare.

## Supplementary Material

RA-016-D6RA07016G-s001

## Data Availability

Data will be available from the authors after the reasonable request. Supplementary information (SI) is available. See DOI: https://doi.org/10.1039/d6ra07016g.
