# Supplementary material for: Mechanically stable X2InAsF6 (X = K, Rb, Cs) for optoelectronic and wasted heat recovery applications
Source: RSC Adv. 2026 Oct 12;16(47):62406–21. doi: 10.1039/d6ra07016g (PMC13656418; doi:10.1039/d6ra07016g)
Supplement: Supplementary file 1 [file RA-016-D6RA07016G-s001.pdf]

Supplementary Information (SI) for RSC Advances.
This journal is © The Royal Society of Chemistry

Mechanically stable X_2InAsF_6 (X = K, Rb, Cs) for optoelectronic and wasted heat recovery applications

Nimra Asif[a], Muhammad Yaseen[a*], Nada Alfryyan[b], Sidra Sarfraz[a], Muhammad Adnan[a], Imed Boukhris[c,d]

[a]Department of Physics, University of Agriculture Faisalabad, Faisalabad 38040, Pakistan

[b]Department of Physics, College of Sciences, Princess Nourah bint Abdulrahman University, P. O. Box 84428, Riyadh 11671, Saudi Arabia

[c]Central Labs, King Khalid University, AlQura'a, Abha, P.O. Box 960,Saudi Arabia)

[d]Department of Physics, Faculty of Science, King Khalid University, P.O. Box 960, Abha, Saudi Arabia

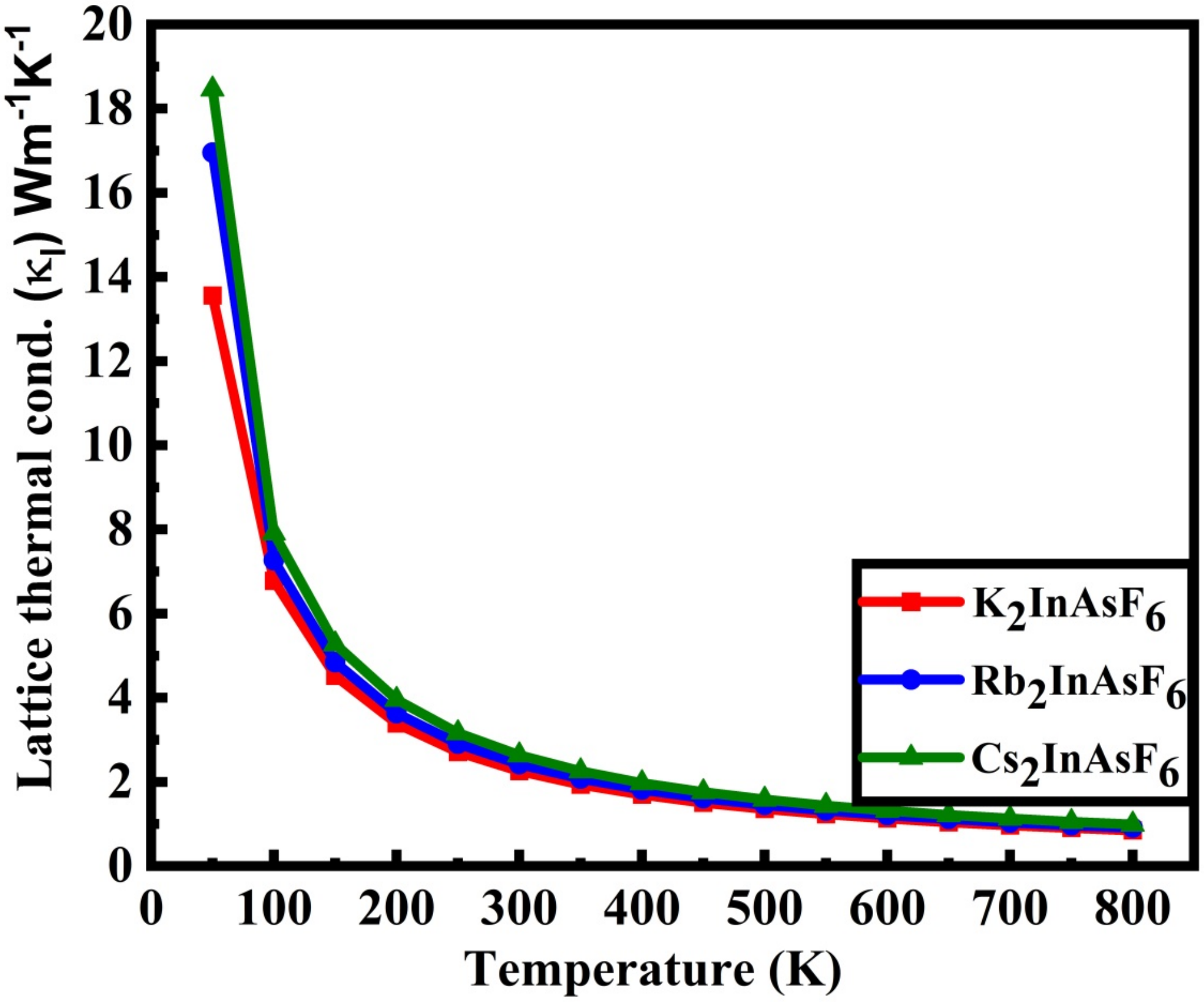

Fig. 1S Lattice thermal conductivity
